# Cardioprotective effect of the *Hibiscus rosa sinensis *flowers in an oxidative stress model of myocardial ischemic reperfusion injury in rat

**DOI:** 10.1186/1472-6882-6-32

**Published:** 2006-09-20

**Authors:** Karunakaran K Gauthaman, Mohamed TS Saleem, Peter T Thanislas, Vinoth V Prabhu, Karthikeyan K Krishnamoorthy, Niranjali S Devaraj, Jayaprakash S Somasundaram

**Affiliations:** 1 Department of Pharmacology, K.M.College of Pharmacy, Madurai-107, India; 2 Department of Biochemistry, University of Madras, Guindy campus, Chennai-600025, India

## Abstract

**Background:**

The present study investigates the cardioprotective effects of *Hibiscus rosa sinensis *in myocardial ischemic reperfusion injury, particularly in terms of its antioxidant effects.

**Methods:**

The medicinal values of the flowers of *Hibiscus rosa sinensis *(Chinese rose) have been mentioned in ancient literature as useful in disorders of the heart. Dried pulverized flower of *Hibiscus rosa sinensis *was administered orally to Wistar albino rats (150–200 gms) in three different doses [125, 250 and 500 mg/kg in 2% carboxy methyl cellulose (CMC)], 6 days per week for 4 weeks. Thereafter, rats were sacrificed; either for the determination of baseline changes in cardiac endogenous antioxidants [superoxide dismutase, reduced glutathione and catalase] or the hearts were subjected to isoproterenol induced myocardial necrosis.

**Results:**

There was significant increase in the baseline contents of thiobarbituric acid reactive substances (TBARS) [a measure of lipid per oxidation] with both doses of *Hibiscus Rosa sinensis*. In the 250 mg/kg treated group, there was significant increase in superoxide dismutase, reduced glutathione, and catalase levels but not in the 125 and 500 mg/kg treated groups. Significant rise in myocardial thiobarbituric acid reactive substances and loss of superoxide dismutase, catalase and reduced glutathione (suggestive of increased oxidative stress) occurred in the vehicle treated hearts subjected to in vivo myocardial ischemic reperfusion injury.

**Conclusion:**

It may be concluded that flower of *Hibiscus rosa sinensis *(250 mg/kg) augments endogenous antioxidant compounds of rat heart and also prevents the myocardium from isoproterenol induced myocardial injury.

## Background

Reperfusion of the ischemic myocardium is the only logical approach for the successful management of patients with IHD. Morphological observations of the ischemic myocardial tissue undergoing reperfusion suggest that it is a true pathological phenomenon and a distinct entity from the preceding ischemic injury [1,2]. Reperfusion of ischemic myocardium is accompanied by the development of oxidative stress and the generation of free radicals which plays a major role in the etiopathogenesis of reperfusion injury [3]. The initial investigations of Rona et al [4] demonstrated that the subcutaneous administration of isoproterenol (ISO) in rats produced graded myocardial necrosis ranging from patchy subendocardial necrosis to transmural infarction, while maintaining a patent coronary vasculature. The rat model of ISO induced myocardial alterations also resulted in a dose dependant manner [5,6]. The process of remodeling a ventricle with diffused myocardial injury and a patent coronary circulation would also be progressive and similar to the observed a discrete myocardial injury due to coronary ligation. The exact mechanism of ISO induced myocardial injury has not been clarified, but a mismatch of oxygen demand versus supply in the presence of coronary vasculature [7] leads to the present investigation related with oxidative stress (induced by isoproterenol) in *in-vivo *model.

The flowers of *Hibiscus rosa sinensis (Fam: Malvaceae) *[HRS], has been reported in the ancient Indian medicinal literature with beneficial effects in heart diseases [8]. In recent times, both experimental and clinical studies have shown that the dried flower powder of HRS has significant protective effects in ischemic heart disease [IHD] [9,10]. However, the exact mechanism of its cardioprotective effects, in respect to the present knowledge of the pathophysiology of IHD, is not well investigated. The present study was designed to investigate the effects of the chronic oral administration of the flower powder of *Hibiscus rosa sinensis *on the endogenous antioxidant status and on oxidative stress induced by isoproterenol in an *in-vivo *model of ischemic reperfusion injury of heart.

## Methods

### Plant material

The flowers of *Hibiscus rosa sinensis *were obtained from the Southern part of India (Thriuvallur District, Tamil Nadu), and the Pharmacognostic authentication was done by the Department of Plant Sciences, University of Madras [vide voucher no 035].

#### Preparation of Hibiscus rosa sinensis suspension

The flower was air dried under shade and pulverized. A suspension of the flower in 2% carboxymethyl cellulose (Vehicle) was made daily.

### Experimental procedure

Male Wistar albino rats weighing 150–200 g were obtained from the Tamil Nadu Veterinary and Animal Science University and were housed at 25° ± 5°C under 12 hour light and dark cycle. Experiments were carried out according to the guidelines of the animal ethics committee of the Institute. The rats were divided into 4 groups (20 in each group) and fed either with the suspension of dried pulverized *Hibiscus rosa sinensis *flower powder of three doses (125 mg/kg (H1), 250 mg/kg (H2) and 500 mg/kg (H3)] or with vehicle by oral gavage once a day for 4 weeks (6 days/week), along with standard rat chow (Amrut Laboratory Animal feed, Bangalore contains protein 22.06%, fat 4.28%, fibre 3.02%, ash 7.8 %, sand [silica] 1.37% w/w) and water, ad libitum. There was no significant difference in body weight of the treated rats, when compared with control, either at the beginning or at end of the study period. The treated rats did not offer any abnormal resistance to drug administration. The treatment schedule did not cause any change in food and water intake pattern. After 48 hours of the last dose, rats were heparinised [375 units/200 g i.p] [11] and half an hour later rats were anaesthetized with anaesthetic ether, and subjected to any one of the protocols

#### Protocol I

Hearts from ten rats of each group were harvested and stored in liquid nitrogen for estimation of basal endogenous antioxidants and in 10% buffered formalin for histological studies.

The groups studied were

**Group control**: Vehicle treated rats

**Group H1 BL**: 125 mg/kg of *Hibiscus rosa sinensis *treated rats

**Group H2 BL**: 250 mg/kg of *Hibiscus rosa sinensis *treated rats

**Group H3 BL**: 500 mg/kg of *Hibiscus rosa sinensis *treated rats

#### Protocol II

At the end of the treatment period 6 rats from each group were administered isoproterenol (ISO) 85 mg/kg s.c., for two consecutive days to induce myocardial injury [4,12]. After 48 hours of the first dose of isoproterenol the rats were sacrificed, hearts were harvested and immediately frozen in liquid nitrogen for biochemical estimation and in 10% buffered formalin for histological studies.

**Group control: **Vehicle + saline injected rats

**Group IR : **Vehicle + ISO treated rats

**Group H1 IR: **125 mg/kg of *Hibiscus rosa sinensis *+ ISO treated rats

**Group H2 IR: **250 mg/kg of *Hibiscus rosa sinensis *+ ISO treated rats

**Group H3 IR: **500 mg/kg of *Hibiscus rosa sinensis *+ ISO treated rats

### Biochemical parameters

#### Myocardial Thiobarbituric acid reactive substances [TBARS]

TBARS levels in the myocardium were determined by the method described by Ohkawa et al., [13].

#### Myocardial reduced glutathione [GSH]

Myocardial GSH was estimated by the method of Ellman, [14].

#### Myocardial superoxide dismutase [SOD]

SOD levels in the hearts were determined by McCord and Firdovich method (1969) and modified by Kakkar et al [15].

#### Myocardial catalase

Catalase level was estimated by the method described by Aebi, [16].

#### Estimation of protein

Protein estimation for the tissue samples were done by the method of Bradford [17].

### Histological examinations

The hearts were removed, washed immediately with saline and then fixed in 10% buffered formalin. The hearts stored in 10% buffered formalin, were embedded in paraffin, sections cut at 5 μm and stained with hematoxylin and eosin. These sections were then examined under a light microscope for histological changes.

### Statistical analysis

All values were expressed as Mean ± SE. (n = 10 in each groups). One way ANOVA was applied to test for significance of biochemical data of the different groups. Significance is set at p < 0.001.

## Results

### Mortality data

There was no mortality in the *Hibiscus rosa sinensis *treated groups and the *Hibiscus rosa sinensis *treated groups subjected to ISO administration. Three rats died in the vehicle + ISO injected group (**IR)**.

There were no significant changes in the levels of TBARS and antioxidants between the vehicle treated and vehicle + saline injected groups of rats. Hence, the results given in the result portions represent the values of vehicle treated rats as control (C).

#### Baseline changes (Table 1)

**Table 1:** Effect of *Hibiscus rosa sinensis *on TBARS, GSH, SOD and catalase in rat heart

**PARAMETERS**	**Control**	**IR**	**H1 BL**	**H1 IR**	**H2 BL**	**H2 IR**	**H3 BL**	**H3 IR**
**TBARS** nmole/g wet wt	46.2 ± 1.5	67.8 ± 3.4*	48.8 ± 2.4	74.1 ± 5.3	58.3 ± 2.6*	49.3 ± 1.9	63.6 ± 2.6*	57.8 ± 1.7
**GSH** μg/g wet wt	320.3 ± 6.9	289.7 ± 2.4*	312.8 ± 4.6	279.3 ± 9.8	441.1 ± 2.9*	372.1 ± 2.9	375.3 ± 1.5*	271.2 ± 7.4
**SOD** I.U/mg protein	2.8 ± 0.3	1.5 ± 0.5*	2.7 ± 0.6	2.5 ± 0.8	5.9 ± 0.6 *	4.2 ± 0.7	4.4 ± 0.8*	2.5 ± 1.1
**CAT** I.U/mg protein	43.1 ± 5.3	30.6 ± 2.7	40.7 ± 5.8	37.8 ± 4.5	59.4 ± 1.6*	46.4 ± 4.3	50.1 ± 1.4*	35.6 ± 1.8

All values are expressed as Mean ± SE (n = 6).

*p < 0.001 vs Control (C) p < 0.001 vs IP IR (One way ANOVA)

**Group Control (C) **– Vehicle (saline) treated rats

**Group IR **– Vehicle-treated rat hearts, Saline-fed and 85 mg/kg isoproterenol injection

**Group H1 BL **– Rats treated with 125 mg/kg of flowers of *Hibiscus rosa sinensis*

**Group H1 IR **– 250 mg/kg of flowers of *Hibiscus rosa sinensis *treated rat hearts subjected to 85 mg/kg isoproterenol injection

**Group H2 BL **– Rats treated with 250 mg/kg of flowers of *Hibiscus rosa sinensis*

**Group H2 IR **– 250 mg/kg of flowers of *Hibiscus rosa sinensis *treated rat hearts subjected to 85 mg/kg isoproterenol injection

**Group H3 BL **– Rats treated with 500 mg/kg of flowers of *Hibiscus rosa sinensis*

**Group H3 IR **– 500 mg/kg of flowers of *Hibiscus rosa sinensis *treated rat hearts subjected to 85 mg/kg isoproterenol injection

Baseline changes brought about by pretreatment for 4 weeks (6 days/week) with three different doses of 125 mg/kg (H1BL), 250 mg/kg (H2BL) and 500 mg/kg (H3BL) or vehicle treated groups are given below:

#### Myocardial TBARS (Fig. 1)

**Figure 1:**
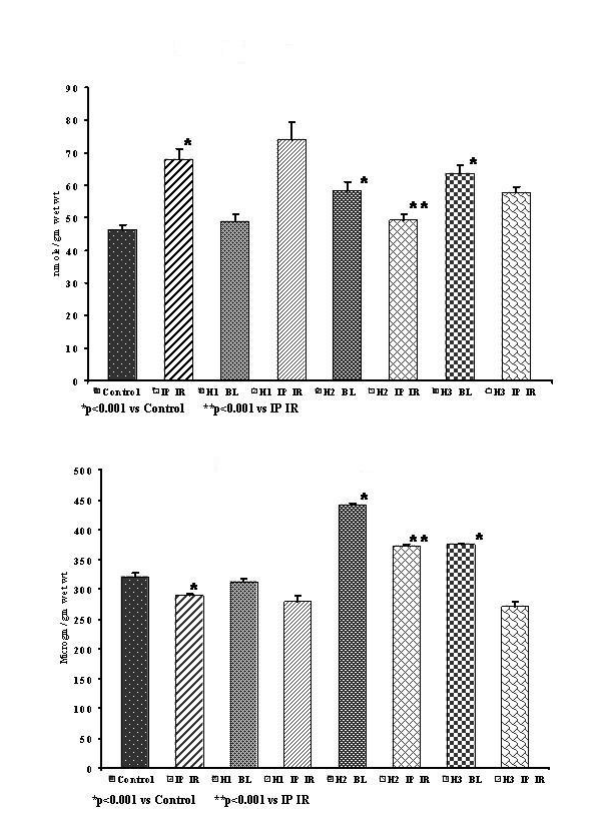
**(A) **Myocardial TBARS. **(B) **Myocardial GSH.

Myocardial baseline TBARS were significantly high (p < 0.001) in H2BL and H3BL groups (58.3 ± 2.6 and 63.6 ± 2.6 nmole/g wet wt), but not in H1BL group (48.8 ± 2.4 nmole/g wet wt) in comparison to control group (46.2 ± 1.5 nmole/g wet wt).

#### Myocardial GSH (Fig. 1)

Myocardial baseline GSH was significantly increased (p < 0.001) in both H2BL and H3BL groups (441.1 ± 2.9 and 375.3 ± 1.5 μg/g wet wt), whereas in H1BL group the GSH level was not significantly altered (312.8 ± 4.6 μg/g wet wt) in comparison to that of control group (320.3 ± 6.9 μg/g wet wt).

#### Myocardial SOD (Fig. 2)

**Figure 2:**
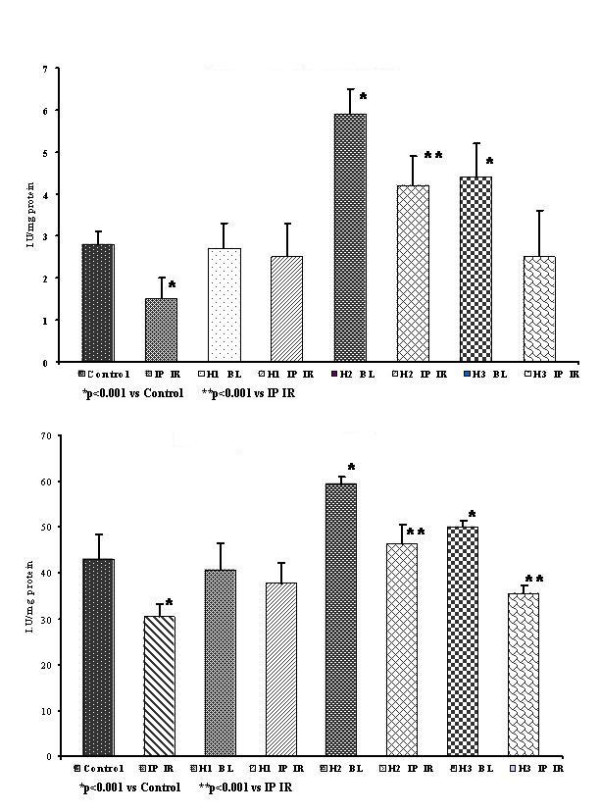
**(A) **Myocardial SOD. **(B) **Myocardial Catalase.

Myocardial baseline SOD was significantly (p < 0.001) increased in H2BL and H3BL groups (5.9 ± 0.6 and 4.4 ± 0.8 I.U/mg protein) in comparison to control group (2.8 ± 0.3 I.U/mg protein). But in H1BL group, there was no significant change (2.7 ± 0.6 I.U/mg protein) in comparison to control group.

#### Myocardial catalase (Fig. 2)

Myocardial baseline catalase was significantly (p < 0.001) increased in H2BL groups (59.4 ± 1.6 I.U/mg protein) whereas in H1BL and H3BL groups the catalase showed no significant change (40.7 ± 5.8 and 50.1 ± 1.4 I.U/mg protein) in comparison to the control group (43.1 ± 5.3 I.U/mg protein).

### Ischemic reperfusion injury – induced changes

The results obtained in the different groups subjected to in-vivo ischemic reperfusion injury are presented below. (Table 1)

#### Myocardial TBARS (Fig. 1)

Myocardial TBARS in IR group (67.8 ± 3.4 nmole/g wet wt) was significantly (p < 0.001) higher than that in control group (46.2 ± 1.5 nmole/g wet wt). In H2 IR and H3 IR treated groups there was a significantly (p < 0.001) lower TBARS (49.3 ± 1.9 and 57.8 ± 1.7 nmole/g wet wt) respectively, whereas in the H1 IR group the TBARS shows no significant change (74.1 ± 5.3 nmole/g wet wt) in comparison to IR group

#### Myocardial GSH (Fig. 1)

Myocardial GSH level was significantly lower (p < 0.001) in IR group (289.7 ± 2.4 μg/g wet wt) in comparison to that of the control group (320.3 ± 6.9 μg/g wet wt). There was a significant increase (p < 0.001) in the levels of GSH in the H2 IR (372.1 ± 2.9 μg/g wet wt), whereas in there was no change in the levels of GSH levels H1 IR (279.3 ± 9.8 μg/g wet wt) and H3 IR (271.2 ± 7.4 μg/g wet wt); in comparison to the IR group.

#### Myocardial SOD (Fig. 2)

Myocardial SOD activity was significantly lower (p < 0.001) in IR group (1.5 ± 0.5 I.U/mg protein) than that in control group (2.8 ± 0.3 I.U/mg protein). Myocardial SOD levels showed no significant change in the H1 IR and H3 IR groups (2.5 ± 0.8 I.U/mg protein and 2.5 ± 1.1 I.U/mg protein respectively) in comparison to IR group. However, the myocardial SOD level was significantly higher (p < 0.001) in the H2 IR group (4.2 ± 0.7 I.U/mg protein) in comparison to IR group.

#### Myocardial catalase(Fig. 2)

Myocardial catalase was significantly lower (p < 0.001) in the IR group (30.6 ± 2.7 I.U/mg protein) in comparison to that of the control group (43.1 ± 5.3 I.U/mg protein). There was no change in myocardial catalase levels in the H1 IR (37.8 ± 4.5 I.U/mg protein) and the H3 IR (35.6 ± 1.8 I.U/mg protein) groups, whereas in H2 IR group myocardial catalase was significantly (p < 0.001) higher (46.4 ± 4.3 I.U/mg protein) in comparison to the IR group.

### Histological changes

**Figure 3:**
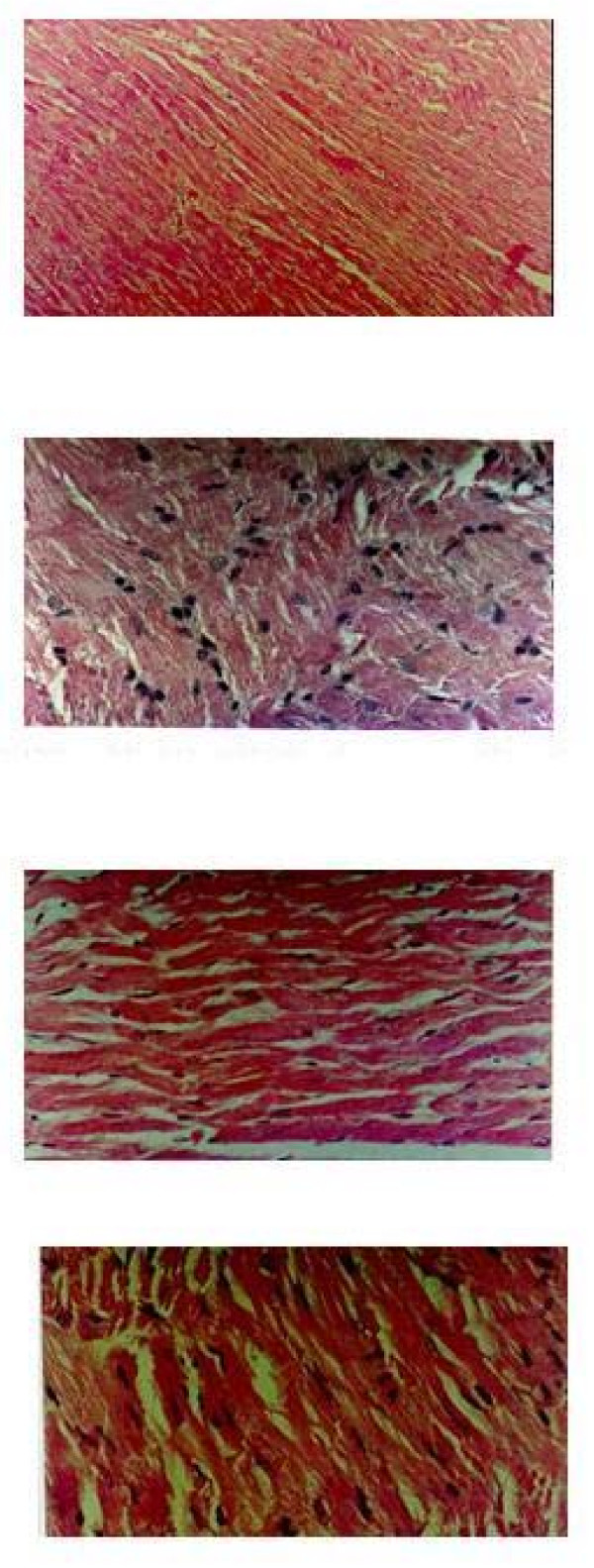
H & E ×400 stained light microscopy section of group **BL **rat myocardium showing well-maintained myofibrillar structure. H & E ×400 stained light microscopy section of group **IR **rat myocardium showing extensive degeneration of myofibrils with leukocytic accumulation, edema and vacuolization. Microscopic section of **H1 BL **treated rat heart. Microscopic section of **H1 IR **treated rat heart H & E ×400.

Light microscopy of the tissue sections of group control showed normal myofibrillar structure with striations, branched appearance and continuity with adjacent myofibrils [Fig 3]. Group IR showed edema, focal haemorrhage and leukocyte infiltration. The muscle fibres showed vascular changes with fragmentation suggestive of necrosis [Fig. 3]. The tissue sections of group H1 BL showed normal myofibrillar structure with striations, branched appearance and continuity with adjacent myofibrils [Fig.3]. Group H1 IR showed hydrophobic changes of myofibrillar structure with striations, branched appearance and continuity with adjacent myofibrils [Fig. 3].

**Figure 4:**
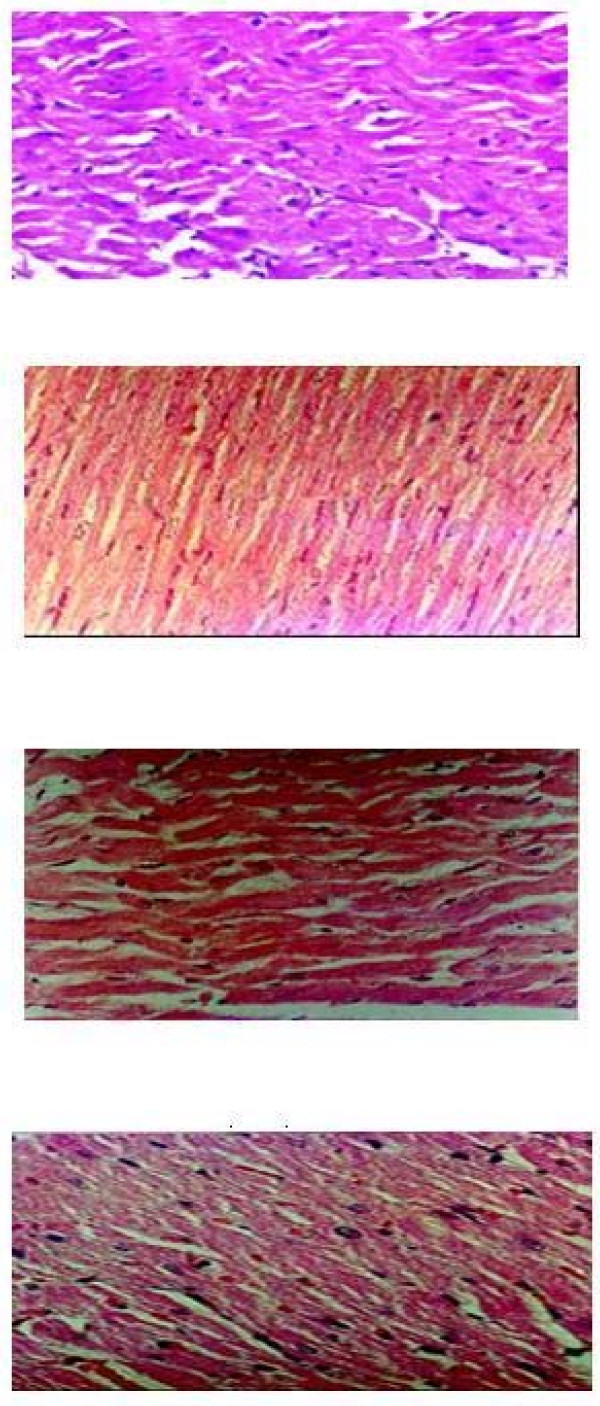
Microscopic section of **H2 IR **treated rat heart H & E ×400. Microscopic section of **H3 BL **treated rat heart H & E ×400. Microscopic section of **H3 IR **treated rat heart H & E ×400.

The group H2 BL showed normal myofibrillar structure with striations, branched appearance and continuity with adjacent myofibrils [Fig. 4]. The H2 IR group showed normal architecture of myofibrillar structure with striations, branched appearance and continuity with adjacent myofibrils. The morphology of cardiac muscle fibres was relatively well preserved [Fig. 4]. The group H3 BL showed mild swelling of myofibrillar structure with striations, branched appearance and no continuity with adjacent myofibrils [Fig. 4]. The tissue sections of group H3 IR showed extensive cellular leukocyte infiltration, marked degeneration of muscle fibres, edema and haemorrhage. [Fig. 4]

## Discussion

In the present study chronic oral administration of *Hibiscus rosa sinensis *flower powder caused significant rise in myocardial TBARS as well as with endogenous antioxidants (SOD, GSH and Catalase) in the 250 and 500 mg/kg treated groups but not with other baseline treated groups. The increase in TBARS is indicative of an enhanced oxidative stress, which in the absence of any evidence of cellular injury (as evidenced by histological studies), may be considered as non-lethal. It is, therefore possible that the increase in oxidative stress was non-lethal and might be responsible for cellular adaptive mechanisms. Recent studies show that various plants and plant extracts can also stimulate the synthesis of cellular antioxidants [18-20]. *Hibiscus rosa sinensis *flowers [10] and Grape [21] contain anthocyanins, which may be responsible for its antioxidant effects. Augmentation of endogenous antioxidants by therapeutic substances has recently evoked scientific interest because any such property of a therapeutic agent can be expected to cause significant improvement in the endogenous defense against oxidative stress [22]. The principal finding of the present study is that ischemic reperfusion injury (IRI) was associated with oxidative stress, as evidenced by increase in myocardial TBARS and depletion of myocardial endogenous antioxidant status (SOD, GSH and catalase). Similar observations were made earlier by other studies [12,23-25]. Chronic oral administration of flowers of *Hibiscus rosa sinensis *prevents the oxidative stress and the structural changes associated with IRI. The mechanism of such protection of chronic oral administration of *Hibiscus rosa sinensis *may be due to myocardial adaptation, oxidative stress is mediated through augmentation of cellular antioxidants such as glutathione, SOD, catalase [26]. In ischemic heart disease, ischemic reperfusion injury is a common sequel and oxidative stress plays a central role in its etiopathogenesis. Protection against oxidative stress through this mechanism may be one of the effective therapeutic approaches. It is developed in various types of stressful conditions, like ischemia, ROS, endotoxins and protects the myocardium from consequent exposure to injuries of similar or more severe in nature [27-29]. Although protective in nature the basic mechanisms of such induction of adaptation are hurtful and therefore, cannot be relied upon as an acceptable therapeutic method. Therefore, identification of the genes, which are important in mediating these effects, has recently become the focus of scientific interest [29-34].

In *in-vivo *ischemic reperfusion injury of the 250 mg/kg treated rat, there was a significant decrease in the TBARS but not in the other treatment groups. Significant rise in the levels of GSH, SOD and catalase were observed in all the treatment groups, and it shows better recovery profile along with histological improvement which, was seen only in the 250 mg/kg treated group subjected to *in-vivo *ischemic reperfusion injury. This indicates that this dosage withstands the oxidative stress associated with *in-vivo *ischemic reperfusion injury. It is particularly noteworthy that both superoxide dismutase and catalase were increased, since it has been shown that an increase in cellular SOD without a concomitant increase in catalase is more harmful by favoring the generation of H_2_O_2 _[22]. The exact mechanism(s) of such a loss of protective effect in higher dose is not clearly understood.

## Conclusion

In this respect, the present study showed for the first time that the flowers of *Hibiscus rosa sinensis *are particularly useful agents, as they could enhance myocardial endogenous antioxidants without producing any cytotoxic effects. Histopathological evidence of myocardial injury following ischemic reperfusion injury was also preserved. Therefore, the protection against myocardial ischemic reperfusion injury in the treated rats is attributed to enhanced endogenous antioxidant activity.

## Abbreviations

Ischemic heart disease (IHD), Isoproterenol (ISO), ischemia and reperfusion (I-R), ischemic reperfusion injury (IRI), Thiobarbituric acid reactive substances (TBARS), reduced glutathione (GSH), superoxide dismutase (SOD).

## Competing interests

The author(s) declare that they no competing interests.

## Authors' contributions

**MS, TP, VP, KK SJ **carried out the experimental work, participated in the sequence alignment, drafted the manuscript and performed the statistical analysis. **ND **conceived the study, and participated in its design and coordination. **KG **participated in the design of the study and helped to draft the manuscript. All authors read and approved the final manuscript.

## Pre-publication history

The pre-publication history for this paper can be accessed here:


